# The molecule of DC-SIGN captures enterovirus 71 and confers dendritic cell-mediated viral *trans*-infection

**DOI:** 10.1186/1743-422X-11-47

**Published:** 2014-03-12

**Authors:** Xiao-Xin Ren, Li Ma, Qing-Wei Liu, Chuan Li, Zhong Huang, Li Wu, Si-Dong Xiong, Jian-Hua Wang, Hai-Bo Wang

**Affiliations:** 1 Jiangsu Key Laboratory of Infection and Immunity, Institutes of Biology & Medical Sciences, Soochow University, Suzhou, China; 2 Key Laboratory of Molecular Virology & Immunology, Institute Pasteur of Shanghai, Chinese Academy of Sciences, Shanghai, China; 3 Center for Retrovirus Research, Department of Veterinary Biosciences, The Ohio State University, Columbus, OH, USA; 4 Department of Microbial Infection and Immunity, The Ohio State University, Columbus, OH, USA

**Keywords:** Enterovirus 71, Dendritic cells, Viral *trans*-infection

## Abstract

**Background:**

Enterovirus 71 (EV71) is the main causative agent of hand, foot and mouth disease that occurs in young children. Neither antiviral agents nor vaccines are available for efficiently combating viral infection. Study of EV71–host interplay is important for understanding viral infection and developing strategies for prevention and therapy. Here the interactions of EV71 with human dendritic cells were analyzed.

**Methods:**

EV71 capture, endocytosis, infection, and degradation in monocyte-derived dendritic cells (MDDCs) were detected by Flow cytometry or real-time (RT-) PCR, and MDDCs-mediated EV71 *trans*-infection of RD cells was determined via coculture system. Cell morphology or viability was monitored with microscopy or flow cytometry. SiRNA interference was used to knock down gene expression.

**Results:**

MDDCs can bind EV71, but these loaded-EV71 particles in MDDCs underwent a rapid degradation in the absence of efficient replication; once the captured EV71 encountered susceptible cells, MDDCs efficiently transferred surface-bound viruses to target cells. The molecule of DC-SIGN (DC-specific intercellular adhesion molecule-3 grabbing nonintegrin) mediated viral binding and transfer, because interference of DC-SIGN expression with specific siRNAs reduced EV71 binding and impaired MDDC-mediated viral *trans*-infection, and exogenous expression of DC-SIGN molecule on Raji cell initiated viral binding and subsequent transmission.

**Conclusion:**

MDDCs could bind efficiently EV71 viruses through viral binding to DC-SIGN molecule, and these captured-viruses could be transferred to susceptible cells for robust infection. The novel finding of DC-mediated EV71 dissemination might facilitate elucidation of EV71 primary infection and benefit searching for new clues for preventing viruses from initial infection.

## Background

Enterovirus 71 (EV71) and coxsackievirus A16 (CVA16) belong to the *Picornaviridae* family, and they are the main causative agents of hand, foot and mouth disease in young children [1-5]. There is no antiviral agent available and vaccine development is still ongoing [6-12]. EV71 invades mainly via the oropharyngeal or intestinal mucosa [13-15]. The initial viral expansion mainly occurs in lymphoid tissues and regional lymph nodes. The produced viruses circulate in the blood, and after replication in reticuloendothelial tissues, liver and spleen, they give rise to viremia and onset of clinical features [13-15]. However, which host cells in the mucosa are primarily targeted by EV71, for mediating viral capture, spread and initial infection, remains poorly understood.

Several host molecules have been recently reported to mediate EV71 binding and infection. P-selectin glycoprotein ligand (PSGL)-1 and scavenger receptor class B, membrane 2 (SCARB2) serve as EV71 functional receptors [16-22]. SCARB2 is mainly expressed in intracellular endosomes and lysosomes and can also be used by CVA16 for infection [16]. Sialic-acid-linked glycans, abundantly expressed by respiratory and gastrointestinal epithelia cells, are reported to be used by EV71 for infection of intestinal cells [4]. Human annexin II protein can bind to capsid protein VP1 for enhancing EV71 infectivity [23]. Additionally, dendritic cell (DC)-specific intercellular adhesion molecule-3 grabbing nonintegrin (DC-SIGN) is another molecule that has been found to mediate partially EV71 entry into DCs [24]. Enteroviruses may retain strain-specific usage of diverse receptors for infection [25].

DCs are pivotal immune cells in modulating host immunity, however, they can be hijacked by several viruses for spread [26-28]. The interactions between DCs and viruses play a key role in viral pathogenesis. However, few studies have investigated the interaction between EV71 and DCs. Here, we show that DCs capture EV71 through viral binding to DC-SIGN, and these surface-bound infectious particles can be transferred to encountered susceptible cells for replication amplification. The understanding of DC and EV71 interplay could facilitate the elucidation of EV71 primary infection and benefit the search for new strategies for combating viral infection.

## Results

### MDDCs can efficiently bind EV71

**Figure 1:**
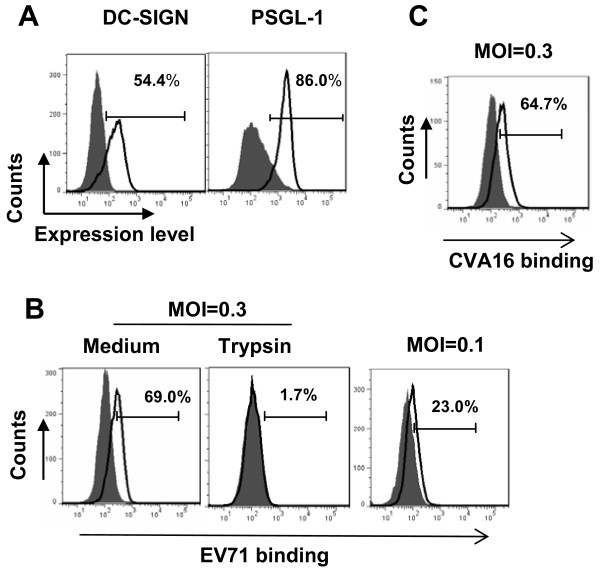
**EV71 viruses bind to MDDCs. (A)** Expression of EV71 receptors or attachment factors in MDDCs. MDDCs cells were immunostained and measured with flow cytometry for expression of PSGL-1 and DC-SIGN. The positive expression percentage is noted. **(B**,**C)** Viral binding to MDDCs. MDDCs were incubated with EV71 or CVA16 viruses for 1 h at 4°C (MOI 0.3 or 0.1). Some samples were treated with trypsin. Surface-bound viruses were measured with flow cytometry through immunostaining with anti-VP1 polyclonal antibodies. One representative donor from four with similar results is shown.

Expression of some identified EV71 receptors or attachment factors, PSGL-1 and DC-SIGN was monitored in MDDCs by flow cytometry. PSGL-1 and DC-SIGN were observed for abundant expression on the MDDC surface (Figure 1).

To investigate infection of MDDCs with EV71, viral binding was first assessed. At 4°C, 69% of MDDCs were positive for EV71 binding, as tested with immunostaining of VP1 and quantified by flow cytometry, and the majority of bound viruses were removed from the cell surface with trypsin treatment, and the lower amount of viruses input generated lower viral binding, indicating the dose-dependent effect (Figure 1B). These data demonstrate that MDDCs can efficiently bind EV71 onto cell surface.

CVA16 is another causative agent of HFMD but not causing CNS diseases. We found that MDDCs could also efficiently bind CVA16 (64.7%, in Figure 1C) as much as EV71 (69%, in Figure 1B). Both EV71 and CVA16 can bind MDDCs efficiently, which may suggest that the initially viral attachment is not the key cause for these two kinds of viruses to induce distinct pathogenesis.

### Loaded-EV71 particles in MDDCs undergo a rapid degradation

**Figure 2:**
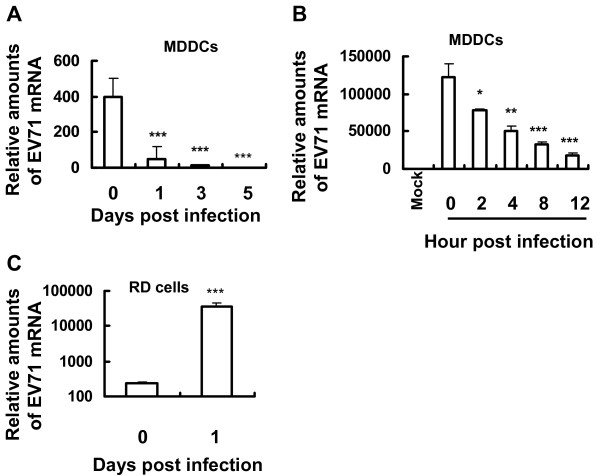
**EV71 uptake and replication.** MDDCs or RD cells were incubated with EV71 for 1 h at 37°C, and then treated with trypsin to remove surface-bound viruses. Viral uptake and replication were quantified by detecting viral RNA level at the indicated time post-infection. **(A**, **C)** MOI 0.1, and **(B)** MOI 1. One representative from three donors with similar results is shown. Data are mean ± SD. ***P <0.001, **P <0.01, and *P <0.05 were considered significant differences in paired *t* test.

We demonstrated above the efficient binding of EV71 to MDDCs. To examine further the potential infection of MDDCs by EV71 in our culture system, varied amounts of infectious EV71 were inoculated to MDDCs at 37°C, and these surface-bound viruses were removed by trypsin treatment. Viral replication was evaluated at the indicated time post-infection by quantifying relatively viral mRNA. No accumulation of EV71 mRNA was observed at 1, 3 and 5 days post-infection (Figure 2A). Instead, EV71 was rapidly degraded in MDDCs, which was assessed through intensive analysis of time-course culture (Figure 2B). As a control, these input EV71 viruses could efficiently and productively infect susceptible RD cells, as viral mRNA was detected 1 day post-infection (Figure 2C). Together, these data indicate that MDDCs do not support efficient replication of EV71; instead, and loaded viruses exhibit a short period of intracellular retention.

### MDDCs can mediate EV71 *trans*-infection

**Figure 3:**
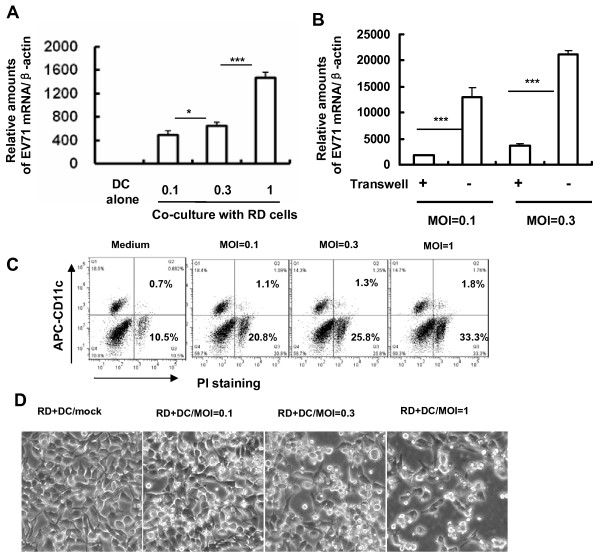
**MDDCs transfer EV71 to susceptible cells. (A)** MDDC-mediated EV71 transmission. MDDCs were incubated with different amounts of EV71 for 1 h at 37°C, after washing, EV71-loaded MDDCs were cocultured or not (DC alone) with RD cells for 1 day. Viral replication was detected by quantifying viral mRNA level. One representative from three donors or repeats with similar results is shown. **(B)** Separation of MDDCs with RD cells abolished EV71 transfer. EV71-loaded MDDCs were separated from RD cells by Transwell insert, and viral transmission was detected as above. **(C**, **D)** Replication of transferred-EV71 killed RD target cells. PI-exclusion staining was used to examine cell death during coculture, and MDDCs (CD11c^+^) and RD cells (CD11c^−^) were distinguished by CD11c staining **(C)**, and cell death was observed under light microscopy **(D)**. Data are mean ± SD. ***P <0.001 and *P <0.05 were considered significant differences in paired *t* test.

DCs can be hijacked by multiple viruses for capture and transfer to susceptible cells for robust infection [26-28]; a process defined as *trans*-infection. To investigate whether MDDCs can mediate *trans*-infection of surface-bound EV71, coculture of virus-pulsed MDDCs with susceptible RD cells was used. As shown in Figure 3A, EV71 could be efficiently transferred from DCs to RD cells for robust infection. Efficient EV71 transfer between cells depended on MDDC and RD cell contact, because viral transfer was significantly inhibited when virus-captured MDDCs were separated from RD cells with Transwell plates (insert membrane size, 0.4 μm) (Figure 3B).

Infection with EV71 can induce apoptosis of multiple types of host cells [29-36]. We demonstrated that robust infection of transferred EV71 also induced killing of RD cells (CD11c-negative), as measured by PI exclusion staining, but MDDC viability (CD11c-positive) was not greatly affected (Figure 3C). Cell death could also be observed from the altered cell morphology (Figure 3D).

Taken together, these data demonstrate that MDDCs can mediate *trans*-infection of surface-bound EV71 upon contact with susceptible RD cells, and robust infection of transferred viruses can induce death of RD target cells.

### DC-SIGN molecule mediates viral binding and *trans*-infection

**Figure 4:**
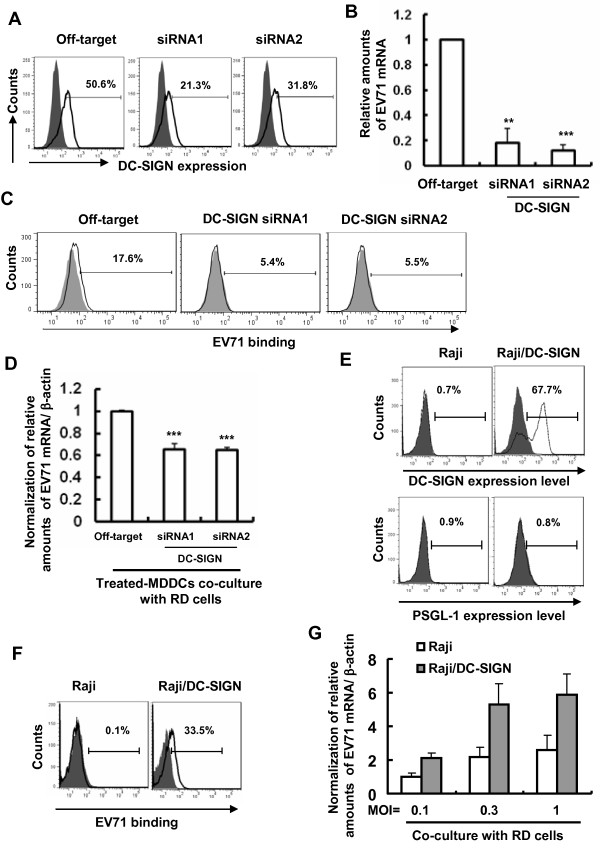
**DC-SIGN molecule mediates viral surface binding and transfer. (A)** Knocking-down of DC-SIGN expression in MDDCs. Expression of DC-SIGN was interfered with by specific siRNAs, and detected by flow cytometry, the positive expression percentage is shown; **(B**, **C)** Interference of DC-SIGN expression impaired EV71 binding as quantified by detecting viral mRNA **(B)** and by measuring with flow cytometry **(C)**. **(D)** DC-SIGN Knocking-down impaired MDDC-mediated EV71 *trans*-infection of RD cells. Data are mean ± SD. ***P <0.001, **P <0.01, were considered significant differences in paired *t* test. **(E**, **F**, **G)** Raji/DC-SIGN cells mediate EV71 binding and transmission. Parental Raji cells and DC-SIGN-expressing derivative were tested for expression of DC-SIGN and PSGL-1 with flow cytometry **(E)**. EV71 was added to cells (MOI 0.3) for viral binding at 4°C, and bound viruses were quantified by flow cytometry **(F)**, and in **(G)** Raji and Raji/DC-SIGN cells were incubated with different amounts of EV71 for 1 h at 37°C, after washing, cells were cocultured with RD cells for 1 day, and viral replication was detected as above. One representative from three repeats is shown.

The molecule of DC-SIGN has been widely described to capture multiple kinds of viruses for subsequent dissemination to susceptible cells [27,28]. To determine the role of DC-SIGN molecule in EV71 binding, expression of DC-SIGN was knocked down with specific siRNA (Figure 4A), and the surface-bound EV71 was quantified. Knockdown of DC-SIGN significantly impaired EV71 binding to MDDCs by detecting viral mRNA (Figure 4B). The bound-viruses also were quantified with immuno-staining with anti-VP1 antibodies, followed with secondary antibodies, and detected with flow cytometry. Knockdown of DC-SIGN decreased the percentage of positive cells, indicating the less viral binding (Figure 4C). Consequently, the knockdown of DC-SIGN also reduced significantly MDDC-mediated EV71 *trans*-infection (Figure 4D).

The role of DC-SIGN in binding and *trans*-infection of EV71 was further evaluated on DC-SIGN-expressing Raji cell derivatives (Figure 4E). No expression of PSGL-1 was observed on either cell type (Figure 4E). Exogenous expression of DC-SIGN on Raji cells facilitated cell surface binding of EV71 virus, when performed at 4°C (Figure 4F). No replication of EV71 in either Raji or Raji/DC-SIGN cells was observed (data not shown). Moreover, these bound-EV71 viruses on Raji/DC-SIGN cells could also be transferred to RD cells (Figure 4G), demonstrating the role of DC-SIGN in mediating EV71 *trans*-infection. Overall, these data demonstrate that DC surface-expressed DC-SIGN mediates EV71 binding and subsequent transfer.

## Discussion

The study of EV71–host interplay is of importance for understanding viral infection and could benefit design strategies for prevention and therapy [37,38]. Here, we show that DCs can transfer surface-bound EV71 to susceptible RD cells for robust infection, although no apparent viral replication in MDDCs was observed. The surface-expressing viral attachment factor DC-SIGN mediates EV71 binding and transfer.

DC-SIGN has been shown to be hijacked by several types of viruses for mediating capture and transmission [27,28]. DC-SIGN can bind high-mannose-containing glycoproteins, such as HIV-1 gp120 and HCV E1 and E2 glycoprotein [27]. DC-SIGN has been demonstrated to mediate partially EV71 binding to MDDCs [24], although the specific molecular basis of EV71 that accounts for binding to DC-SIGN needs to be further clarified.

DC-SIGN also can mediate viral endocytosis [39], and here, we observed EV71 internalization into MDDCs. Whether DC-SIGN plays a role in viral endocytosis needs to be further examined. A recent publication reported that both SCARB2 and PSGL-1 could bind EV71 viruses when these receptors were stably expressed on mouse L929 cells, and SCARB2 efficiently mediated viral internalization and uncoating [18]. Clathrin-mediated endocytosis has been reported to act as an entry pathway for EV71 infection of susceptible RD cells [40]. However, despite EV71 binding and endocytosis into MDDCs, no apparent viral infection was observed, and these internalized viruses were rapidly degraded. The contradiction between EV71 infection of MDDCs in the present and other studies may be due to different viral strains and infection situations [24].

EV71 replication causes apoptosis of a wide range of host cells, such as human glioblastoma cells, microvascular endothelial cell line, neural cells, RD cells, Vero cells, HeLa cells, and Jurkat T cells [29-33,35,36]. Similarly, the replication of transferred EV71 mediated by MDDCs also induced RD cell death in our study, while MDDC viability was not greatly affected. The cell selectivity might have been due to the lower susceptibility of MDDCs for EV71 replication. The pathways of EV71-induced apoptosis remain elusive [34,36,41]. EV71 infection can induce Fas ligand expression on Jurkat T cells [42] and trigger activation of caspase 3 and 8 and poly (ADP-ribose) polymerases in RD cells [36]. Viral protein synthesis appears to be required for inducing cell apoptosis, and expression of 2A and 3C proteases is associated with induction of apoptosis [31,32]. Blocking with specific antibodies such as DC-SIGN prior to EV71 infection could provide an alternative approach for blocking viral replication and viremia by reducing viral binding and transmission mediated by MDDCs.

Overall, although the *in vivo* investigation needs to be further clarified, our *in vitro* findings hint at the potential role of DCs in EV71 primary infection and may benefit the search for new ways to prevent viruses from initial infection.

## Conclusion

In this study, we demonstrated that MDDCs could bind efficiently EV71 viruses through viral binding to DC-SIGN molecule, and these captured-viruses could be transferred to susceptible cells for robust infection. The novel finding of DC-mediated EV71 dissemination might facilitate elucidation of EV71 primary infection and benefit searching for new clues for preventing viruses from initial infection.

## Methods

### Cells

Human peripheral blood mononuclear cells (PBMCs) were purchased from the Blood Center of Shanghai, China. CD14^+^ monocytes were purified from PBMCs using anti-CD14 specific antibody-coated microbeads (Miltenyi Biotec). Monocyte-derived DCs (MDDCs) were generated by stimulation of monocytes with 50 ng/ml granulocyte–macrophage colony-stimulating factor (GM-CSF) (R&D Systems) and interleukin (IL)-4 (R&D Systems) for 5 days, and MDDCs show the phenotype of DC-SIGN^+^CD11c^++^HLA-DR^+^CD14^-/low^CD3^−^CD19^−^. RD cells (rhabdomyosarcoma) (ATCC# CCL-136) were maintained in Dulbecco’s Modified Eagle’s Medium (DMEM; Hyclone) containing 10% fetal bovine serum (FBS; Hyclone) with 100 U/ml penicillin and 100 μg/ml streptomycin. Raji and Raji/DC-SIGN (kind gifts from Dr. Vineet Kewal Ramani, National Cancer Institute, USA) have been described previously [43], and cells were maintained in RPMI 1640 medium containing 10% FBS with 100 U/ml penicillin and 100 μg/ml streptomycin.

### Viral stock

EV71 (G08-2 strain) and CVA16 (SHZH05-1 strain, GenBank^#^ EU262658) were propagated in RD cells as previously described [44,45]. RD cells with 80% confluence were inoculated with EV71 at 37°C for 1 h with occasional shaking, and were incubated after washing in DMEM supplemented with 2% FBS until a 90% cytopathic effect was reached. Cell pellets were harvested and subjected to three freeze–thaw cycles to release the intracellular virus particles. Viral aliquots were stored at –80°C, and virus titers were determined by microtitration using RD cells, which were expressed as the 50% tissue culture infectious dose (TCID50), according to the Reed–Muench method.

### Flow cytometry

Expression of PSGL-1, DC-SIGN and SCARB2 was assessed with flow cytometry. The following antibodies or isotype-matched IgG were used (clone numbers and resources are given in parentheses): PE-anti-DC-SIGN (eB-h209; eBioscience) and purified anti-PSGL-1 monoclonal antibody (KPL-1; BD Pharmingen), followed by staining with secondary anti-mouse antibodies. Stained cells were detected with an LSRII flow cytometer (BD Biosciences Pharmingen) and analyzed with FlowJo 7.6.1 software (TreeStar Inc.).

### Viral binding, endocytosis and infection

For the binding assay, MDDCs or Raji or Raji/DC-SIGN cells (2×10^5^/sample) were incubated with EV71 (4×10^5^ TCID50) for 1 h at 4°C. Some samples were digested with 0.25% trypsin for 5 min. The bound viruses were then stained with VP1 polyclonal antibody, followed by Alexa Fluor 568-goat anti-rabbit IgG (Invitrogen). The anti-VP1 polyclonal antibody was generated by immunization of rabbits with recombinant EV71 VP1 protein produced from *Escherichia coli*, and this antibody was found to cross-react strongly with CVA16 [44]. For viral endocytosis or infection assay, MDDCs or RD cells were inoculated with different amounts of EV71 for 1 h at 37°C; cells were then treated with 0.25% trypsin for 5 min to remove surface-bound viruses, and cultured and harvested at the indicated time. Viral retention or infection was assessed by relative quantification of EV71 mRNA levels.

### Real-time PCR

Total RNAs from differently treated MDDCs or RD cells were extracted by using TRIzol Reagent (Invitrogen), and were reverse transcribed into cDNA by performing the ReverseAid™ First strand cDNA synthesis Kit (Fermentas) according to the manufacturer’s instructions. Two microliters of cDNA reaction was amplified using forward and reverse primers for either EV71 genome or β-actin. Primers were listed as below: EV71-F, 5′-CCCTGAATGCGGCTAATCC-3′, and EV71-R, 5′-ATTGTCACCATAAGCAGCCA-3′; β-Actin-F, 5′-GGGAAATCGTGCGTGACAT-3′, and β-Actin-R, 5′-GTCAGGCAGCTCGTAGCTCTT-3′. Real-time PCR was carried out by using the Thunderbird SYBR qPCR Mix (TOYOBO) with the thermal cycling conditions: initial denaturation at 95°C for 10 min, 40 cycles of denaturation at 95°C for 15 s, primer annealing at 60°C for 15 s, and extension at 72°C for 30 s, followed by final extension at 72°C for 6 min. The level of EV71 RNA was expressed as fold change relative to β-actin.

### EV71 transmission

MDDCs, Raji or Raji/DC-SIGN cells (3 × 10^5^ each) were inoculated with different amount of infectious EV71 for 1 h at 37°C. Cells were washed to remove cell-free viruses and cocultured with RD cells (8 × 10^5^) for an additional 24 h. In some samples, a Transwell plate with an insert membrane size of 0.4 μm was used to separate MDDCs from RD cells. Viral transmission was quantified by detecting EV71 mRNA level relative to β-actin. Cell morphology was observed under a light microscope (Leica), and cell killing was measured by using propidium iodide (PI) (Invitrogen) exclusion staining, and MDDCs and RD cells were distinguished with immunostaining with APC-Alexa Fluor750-CD11c (B-ly6; BD Biosciences Pharmingen).

### siRNA interference

MDDCs were transfected with specific duplex siRNA of DC-SIGN or off-target control (GenePharma). Amaxa human dendritic cell nucleofector kit (Amaxa) was used for transfection according to the manufacturer’s instructions. Cells were harvested to confirm the knockdown of target genes with flow cytometry. Viral binding and transmission were investigated as described above. The sequences of siRNA duplex were listed as below: DC-SIGN siRNA-1:5′-GGAAUGGACAUUCUUCCAATT-3′(sense), 5′-UUGGAAGAAUGUCCAUUCCTT-3′(antisense); DC-SIGN siRNA-2: 5′-GGAUGAAGAACAGUUUCUUTT-3′(sense), 5′-AAGAAACUGUUCUUCAUCCTT-3′ (antisense).

### Statistical analysis

Statistical analysis was performed using a paired *t* test with SigmaStat 2.0 (Systat Software, San Jose, CA, USA).

## Competing interests

The authors declare that they have no competing interests.

## Authors’ contributions

Conceived and designed the experiments: JW, LW, ZH, SX. Performed the experiments: HW, XR, LM, CL. Analyzed the data: HW, XR, JW. Contributed reagents/materials/analysis tools: QL, ZH. Wrote the paper: LW, JW. All authors read and approved the final manuscript.
